# Effects of Non-Enzymatic Browning and Lipid Oxidation on Color of Ready-to-Eat Abalone during Accelerated Storage and Its Control

**DOI:** 10.3390/foods12071514

**Published:** 2023-04-03

**Authors:** Yingchen Fan, Manman Yu, Deyang Li, Guanhua Zhao, Min Zhang, Zonghan Wang, Yuxin Liu, Dayong Zhou

**Affiliations:** 1 School of Food Science and Technology, Dalian Polytechnic University, Dalian 116034, China; 2 College of Tea & Food Science and Technology, Anhui Agricultural University, Hefei 230036, China; 3 National Engineering Research Center of Seafood, Dalian 116034, China; 4 Collaborative Innovation Center of Seafood Deep Processing, Dalian 116034, China

**Keywords:** ready-to-eat abalone, color, lipid oxidation, accelerated storage

## Abstract

The deepening of color of ready-to-eat (RTE) abalone during storage leads to sensory quality degradation, which seriously affects the shelf life of products and consumers’ purchasing desire. The goal of this study is to look into the causes of non-enzymatic browning and lipid oxidation, as well as how to control them, and their effect on the color of RTE abalone during storage. The control, bloodletting and antioxidants groups (lactic acid, citric acid and 4-hexylresorcinol) of RTE abalone were stored for 0, 20 and 40 days at 40 °C, respectively, to explore the rule and mechanism of the color change in RTE abalone. This research shows that RTE abalone undergoes browning during storage. Meanwhile, the content of reducing sugar, phenols and unsaturated fatty acids decreases, while the formation of lipid hydroperoxides and aldehydes increases during storage. In addition, the color change in RTE abalone during storage is mainly related to the Maillard reaction, while the lipid oxidation mainly forms pyrrole and participates in the Strecker degradation process as part of the Maillard reaction. The quality of RTE abalone can be maintained by controlling browning effectively as well as lipid oxidation through bloodletting and the addition of antioxidants to ensure that RTE abalone has high storage stability. According to our research, bloodletting and the addition of antioxidants to RTE abalone have a good application prospect and popularizing value in the storage of RTE abalone.

## 1. Introduction

Abalone (*Haliotis discus hannai Ino*) is a single-shell shellfish with high economic value and a global production of 21,000 tons in 2020 [1]. The gastropod muscle of abalone, which is the edible part, is a popular delicacy due to the soft, chewy and elastic texture as well as unique flavor of cooked abalone [2]. At present, processed abalone in the form of dried and canned products is widely available on the market because of its long shelf life [3]. Ready-to-eat (RTE) seafood is soft canned food that has been cooked, packed using soft vacuum, sterilized and stored at room temperature. [4]. Recently, because of easy handling, eating and storing, RTE seafood has become popular with consumers [5]. However, RTE abalone will undergo some changes during room-temperature storage, such as flavor deterioration, texture softening, browning, etc. Among them, color changes seriously affect the shelf life of the product and most likely influence the consumers’ purchasing desire [6]. Moreover, high level of Cu^2+^ in abalone blood may lead to the browning of abalone more easily than other seafood. Therefore, the color preservation of RTE abalone during storage is an industry challenge to be solved.

The color of a freshly cooked abalone muscle is golden yellow, which makes it popular with consumers [7]. After the period of storage, the color of cooked abalone muscle changes from golden yellow to black-brown according to the preliminary experiments, resulting in the deterioration of the sensory characteristics. According to whether the reaction requires the participation of enzymes, the browning reaction can be divided into enzymatic browning and non-enzymatic browning [8]. Obviously, the browning of RTE abalone muscle is mainly non-enzymatic, because it is produced through heat treatment such as cooking and sterilizing which deactivate enzymes. Researches have investigated the browning of cooked shrimp [9], cod [10] and grass carp [11] caused by non-enzymatic browning. However, to the best of our knowledge, the browning of cooked abalone muscle during storage and its mechanism have not been studied.

The blood in abalone contains hemocyanin, which is bounded to two copper ions [12,13]. The copper ions promote the autoxidation of phenolic substances, which may lead to the accelerated browning of RTE abalone. Meanwhile, the effect of pH on non-enzymatic browning of abalone muscle is the most significant [14], and 4-hexylresorcinol (4-HR) is an antioxidant and browning inhibitor which can inhibit browning [15,16]. Therefore, the effects of browning inhibitor (lactic acid, citric acid and 4-HR) and browning accelerator (abalone blood) on browning of abalone muscle were investigated in this study. To achieve this goal, the color of RTE abalone muscle during storage was measured. Meanwhile, the mechanism of color changes during storage was clarified by fatty acid composition, formation of aldehydes and lipid hydroperoxides (POV), as well as reducing sugar content, total phenols content, and the Maillard reaction parameters including Maillard intermediate products, browning index, 5-hydroxymethylfurfural (5-HMF) content and advanced glycation end products (AGEs) fluorescence intensity, in order to study and regulate the color deterioration rule of RTE abalone during storage and to provide a basis for improving the processing technology and quality stability of RTE abalone.

## 2. Materials and Methods

### 2.1. Materials and Chemicals

Fresh abalones (average weight was about 30 ± 2 g) were from a local market in Dalian, Liaoning, China. The 4-hexylresorcinol, food-grade lactic acid and citric acid were from Shanghai Ruixiang Biological Technology Co., Ltd. (Shanghai, China).

### 2.2. Sample Treatment

Forty-five abalones were evenly divided into three groups. For the bloodletting group, the abalone muscle was placed on ice for 1 h, then washed with warm water (45 °C) for 10 min, then the black film was removed, and the muscle was soaked in distilled water (1:4, *w*/*v*) at 4 °C for 3 h. After bloodletting and washing, the abalone muscle in the antioxidants group was soaked in a soaking solution (1:4, *w*/*v*) containing 0.2% LA, 0.2% CA and 4-HR (1 mg/kg) at 4 °C for 3 h. The preparation of the control group was the same as that of the bloodletting group, but without the step of bloodletting on ice. Then each group of abalones was boiled in the corresponding solution at 90 °C for 10 min, dried at 60 °C for 20 min, and then the abalone muscles were packed and sterilized in boiling water for 30 min. Then they were stored at 40 °C for 0, 20, and 40 days, respectively, and later collected as experimental samples. Some of the abalones were directly used in the follow-up experiment, and the rest were freeze-dried for the follow-up experiment.

### 2.3. Color Measurement

The color of abalone muscle was measured by a colorimeter according to the generally accepted CIELAB color scale [17]. The adductor muscles on the back of the abalone muscle were selected for the determination of color. Color was expressed in L* (brightness), a* (+a, redness; −a, greenness) and b * (+b, yellowness; −b, blueness). The formula for calculating whiteness (W*) and chromatic aberration (ΔE) is as follows [17]:

W∗=100 − [(100 − L∗)2+a∗2+b∗2]1/2

ΔE=(ΔL∗2+Δa∗2+Δb∗2)1/2

### 2.4. Determination of Lipid Oxidation

#### 2.4.1. Determination of Oil-Extraction Rate

Using the modified Folch procedure [18] based on our previous study [19], total lipid was extracted from 5 g of dried powder of abalone muscle.

#### 2.4.2. Determination of Fatty Acid Composition

Quantitative analysis of fatty acids was carried out by Agilent GC-MS system as described previously [20]. As a dry basis, the contents of each fatty acid were calculated and expressed as mg/g.

#### 2.4.3. Determination of Lipid Hydroperoxides

The determination method of lipid hydroperoxide was as follows [21]. In short, the oil of abalone (0.2 mL) was mixed with 2.8 mL of 1-butanol/methanol mixture (1:2, *v*/*v*) with subsequent addition of 15 μL of 3.94 mol/L NH_4_SCN solution and 15 μL of freshly configured Fe^2+^ solution. Reaction in the dark for 20 min led to the absorbance of the solution at 510 nm. The calibration curve for measuring hydrogen peroxide was used to determine the concentration of hydrogen peroxide.

#### 2.4.4. Determination of Aldehydes Formation

The formation of aldehydes was determined by the following methods of Zhao et al. [22]. The LC separation was performed on a Shimadzu LC-30AVP system (Shimadzu Co., Tokyo, Japan). Chromatographic separation was achieved by using Zorbax SB-C18 column (4.6 × 50 mm, 1.8 μm, Agilent Technologies, Santa Clara, CA, USA) at 30 °C, 0.3 mL/min flow rate, and 6 μL injection volume. Mobile phase A consisted of 0.1% (*v*/*v*) formic acid in water. The mobile phase B consisted of 0.1% (*v*/*v*) formic acid in acetonitrile. The triple quadrupole mass spectrometer API 5500 Qtrap from AB Sciex was used for the MS detection (Foster City, CA, USA).

### 2.5. Determination of Total Polyphenol Content

Total polyphenol contents were measured as described previously in [23] with some modification. Briefly, 20 mL of an ethanol/water mixture (7/3, *v*/*v*) was combined with 2 g of powdered abalone muscle. The supernatant was gathered and refilled to 25 mL following centrifugation at 3450× *g* for 10 min at 4 °C. Then, 0.5 mL of Folin–Ciocalteu reagent was carefully mixed with 0.5 mL of supernatant, 0.5 mL of distilled water and 1 mL of 10% Na_2_CO_3_ solution (Solarbio, Beijing, China). Following two hours of dark incubation, the solution’s absorbance was measured at 760 nm with a TECAN Infinite M200 microplate reader (Tecan, Mannedorf, Switzerland). The polyphenol content (μg/g) was then measured by gallic acid corresponding to the standard curve.

### 2.6. Determination of Maillard Reaction Products (MRPs)

#### 2.6.1. Determination of Advanced Glycation End Products (AGEs) Fluorescence Intensity

The AGEs fluorescence intensity of abalone muscle was determined using the method of [24]. Briefly, 5 g of abalone muscle was homogenized with 25 mL of deionized water, then centrifuged at 5000× *g* for 10 min. The supernatant and equal volume of 20% TCA were mixed and centrifuged at 5000× *g* for 10 min; the supernatant was collected. The fluorescence intensity of supernatant was determined at excitation and emission wavelengths of 370 nm and 440 nm, respectively.

#### 2.6.2. Determination of 5-Hydroxymethylfurfural (5-HMF) Content

Measurement of the 5-HMF content of abalone muscle was performed as described previously in [25]. The separation and analysis were carried out by a HPLC system with a diode array detector (DAD) monitored at 285 nm. Using an Elite Hypersil C18 column (250 mm 4.6 mm 5 μm) at 30 °C, the samples (10 L) were separated. At 0.6 mL/min isocratic elution, methanol/water (10/90, *v*/*v*) was used as the mobile phase. The 5-HMF contents were calculated from the 5-HMF standard curve, and the contents were expressed as ng/g of abalone muscle.

#### 2.6.3. Determination of Maillard Intermediate Product and Browning Index

The Maillard intermediate product and browning index (BI) of abalone muscle were monitored as reported in [26]. Briefly, 5 g of abalone muscle was homogenized with 25 mL of deionized water, then the homogenate was centrifuged at 5000× *g* for 10 min. The absorbance of supernatant was measured spectrophotometrically at 294 and 420 nm.

### 2.7. Fatty Acid Composition

The reducing sugar content was measured by the method of 3,5-dinitrosalicylic acid (DNS) as described previously in [27] with some modification. Briefly, 2 g of abalone muscle powder was homogenized with 20 mL of distilled water. After centrifugation at 5000× *g* for 10 min at 4 °C, the supernatant was collected and replenished to 25 mL, then 4 mL of distilled water was added to terminate the reaction. Based on the glucose standard curve, the reducing sugar content was calculated based on the absorbance at 520 nm.

### 2.8. Statistical Analysis

All experiments were performed in triplicate. SPSS 19.0 (SPSS Inc., Chicago, IL, USA) was used for statistical analysis, Duncan’s method was used for differential significance analysis, and *p* < 0.05 indicated significant difference. The data chart was made by using Origin 2022 software. Gephi (Version 0.9.2) software was used to visualize the correlative network. The results were shown as mean ± standard deviation.

## 3. Results and Discussion

### 3.1. Appearance and Color of RTE Abalone Muscle during Storage

#### 3.1.1. Appearance

**Figure 1:**
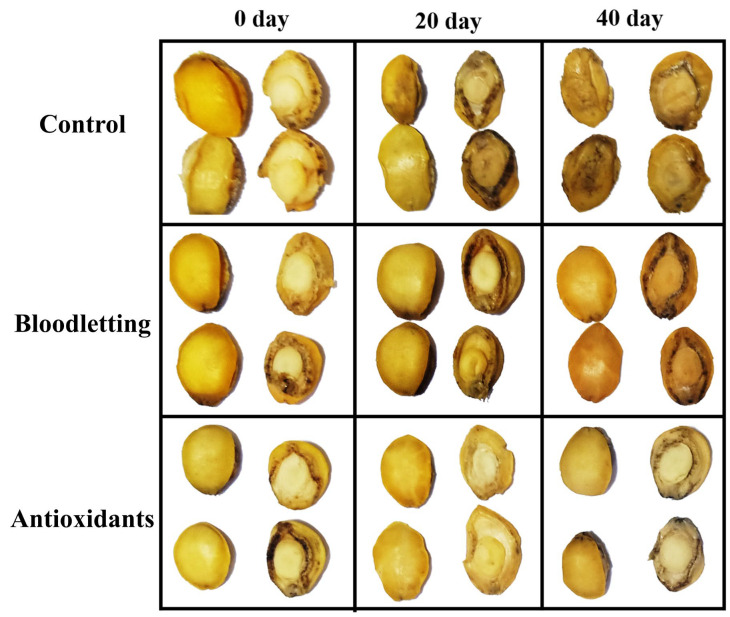
The appearance of abalone muscle during storage.

The appearance of abalone muscle after 0, 20, 40 days is shown in Figure 1. For the three groups, the color of abalone muscle (0 day) was attractive golden yellow, which gradually became darker along with the extension of storage time. After the period of storage, the browning of the bloodletting group was less significant than that of the control group, and the browning of the antioxidants group was less significant than that of the others.

#### 3.1.2. Color

The changes in L*, a*, b*, W* and ΔE values of abalone muscle during storage are shown in Table 1. The L* and W* values represent brightness and whiteness, which were decreased with the extension of storage time. The a* and b* values are generally used to describe redness and yellowness, respectively. For the bloodletting and control group, the a* value first decreased briefly and then rose along with the extension of storage time. At the same time, for the antioxidants group, the a* value increased slowly during storage. For all the three groups, the b* value was stable during storage. The ΔE value, which refers to the test unit of color difference perceived by human eyes in a uniform color perception space, was increased with the extension of storage time. These changes included L*, a*, b*, W* and ΔE values, suggesting that the color of abalone muscle changed from golden yellow to dark brown after storage. By contrast, the order of changes in the L*, W* and ΔE values in different groups was as follows: control group, bloodletting group and antioxidants group. This was consistent with the results of visual perception (Figure 1).

A large number of studies have demonstrated that lipid oxidation and non-enzymatic browning are the main causes of color degradation in aquatic products. In our study, compared to the control group, the bloodletting treatment delayed browning. The blood in abalone contains hemocyanin which is bound to two copper ions [12,13]. Therefore, it is speculated that the copper ions in the blood promote the oxidation of lipids and phenolic substances, leading to accelerated browning in the abalone muscle of the control group. Moreover, the above results also indicate that the addition of antioxidants in the corresponding group significantly inhibited the browning of the abalone muscle during storage. Li et al. [28] reported that phytic acid (PA) and LA treatment could delay the color deterioration of cooked shrimp during storage by effectively slowing down the lipid oxidation and Maillard reaction.

### 3.2. Lipid Oxidation in RTE Abalone Muscle during Storage

#### 3.2.1. Lipid Extraction Rate

**Figure 2:**
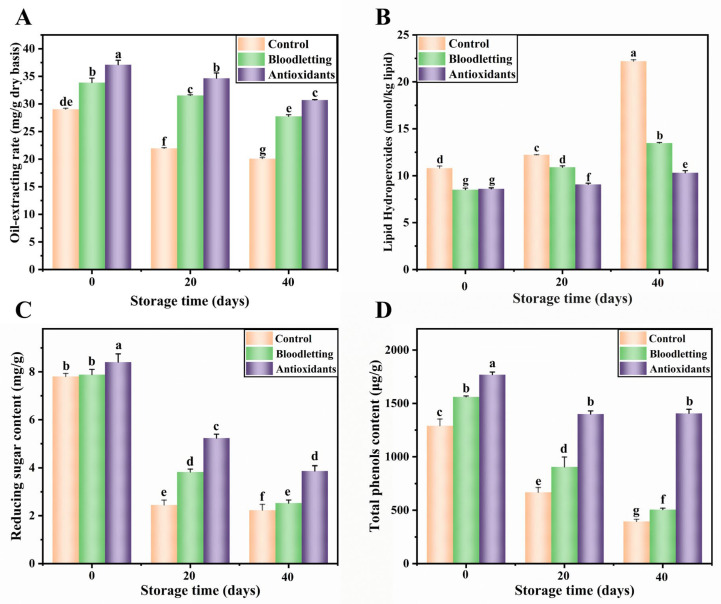
Changes in oil-extraction rate (**A**), lipid hydroperoxides (**B**), reducing sugar content (**C**) and total phenols content (**D**) of abalone muscle during storage. Values of different groups with different lower-case letters are significantly different at *p* < 0.05.

During storage, the lipid extraction rate of RTE abalone showed a down trend (Figure 2A). The initial storage values of lipid extraction rate in the control group, bloodletting group and antioxidants group were 29.04 ± 0.18, 33.86 ± 0.82 and 37.09 ± 0.81 mg/g, respectively, which dropped to 20.08 ± 0.12, 27.76 ± 0.29 and 30.71 ± 0.07 mg/g after 40 days of storage. The decrease in oil extraction rate was mainly due to the decomposition of lipids into small molecules through primary and secondary oxidation reactions during lipid oxidation.

#### 3.2.2. Fatty Acid Composition

The changes in fatty acid contents of RTE abalone muscle are shown in Table 2. The main fatty acids in RTE abalone were linoleic acid (C18:2 n-6), palmitoleic acid (C16:1), tricosanoic acid (C23:0), arachidic acid (C20:0), arachidonic acid (C20:4 n-6) and docosahexaenoic acid (DHA, C22:6 n-3).

Before storage, the contents of monounsaturated fatty acids (MUFAs) and polyunsaturated fatty acids (PUFAs) in the control group were significantly lower than those in the antioxidants group, indicating that adding antioxidants produced an antioxidant protection effect on unsaturated fatty acids (UFAs) during heat treatment. For each group of samples, the contents of saturated fatty acids (SFAs) of RTE abalone were nearly constant, while the contents of MUFAs and PUFAs decreased significantly along with the extension of storage time, indicating that UFAs were oxidized during storage. After the period of storage, the order of UFA contents in RTE abalone was as follows: control group, bloodletting group and antioxidants group. After 40 days of storage, the contents of MUFAs and PUFAs decreased by 18.14% and 22.25%, respectively, for the control group, while the two values were 11.48% and 21.29% for the bloodletting group and 8.91% and 11.13% for the antioxidants group. Uematsu et al. believed that the increase in the unsaturation degree of fatty acids led to the increase of non-enzymatic browning reaction [29]. The results indicate that bloodletting and adding antioxidants produce a significant antioxidant protection effect on UFAs in RTE abalone during storage. Our research team found a similar pattern in the storage of freeze-dried scallops, and the PUFAs in dried scallop adductor muscle can be protected by adding phenols [20].

#### 3.2.3. Lipid Hydroperoxide Value

The lipid hydroperoxide value (POV) can reflect the content of hydroperoxide formed as the primary oxidation product during lipid oxidation [30]. The POV of the three groups increased in a time-dependent manner during the 40 days of storage (Figure 2B). Before storage, the POV of the control group was significantly higher than that of the other two groups, indicating that bloodletting and adding antioxidants can effectively inhibit lipid oxidation during heating. After 40 days of storage, the POV of the control group increased by 105.44%. However, the POV of the bloodletting group and antioxidants group increased by 58.23% and 19.93%, respectively. Cyprian et al. reported that lipid oxidation products such as hydroperoxide and malondialdehyde may react with amines, amino acids or proteins, leading to browning of oily foods [31]. Obviously, the lipid primary oxidation product in RTE abalone was effectively inhibited by bloodletting and adding antioxidants, with the antioxidants group having the best inhibitory effect. Our team found a similar pattern during accelerated storage of dried shrimp (*Penaeus Vannamei*) [32].

#### 3.2.4. Aldehydes Content

The PUFAs are easily oxidized and degraded to form volatile secondary oxidation products such as aldehydes. In this study, propanal, heptanal and trans,trans-2,4-Decadienal were selected for the analysis to represent the secondary oxidation level of RTE abalone during storage according to a previous study [22] (Table 3). Before storage, the contents of these aldehydes in the control group were significantly higher than those in the antioxidants group. The three aldehydes were continuously generated during storage. After the period of storage, the order of contents of aldehydes was as follows: antioxidants group, bloodletting group and control group. Thus, the addition of antioxidants can prevent lipid oxidation and effectively control the formation of aldehydes.

### 3.3. Total Phenols Content in RTE Abalone Muscle during Storage

The changes in total phenols content in abalone muscle during storage are shown in Figure 2D. Before storage, the control group, bloodletting group and antioxidants group contained 1290.62 ± 63.33, 1560.99 ± 8.79 and 1769.33 ± 24.97 μg/g of total phenols, respectively. After being stored for 40 days, the corresponding values dropped to 395.06 ± 20.026, 506.48 ± 13.29 and 1406.45 ± 38.96 μg/g, respectively. Phenolic substances can produce lipid derivatives and quinones by providing hydrogen atoms to lipid free radicals. Then quinones undergo an oxidative cyclization and polymerization reaction, first forming a red intermediate and finally a dark brown substance. Therefore, the oxidation of phenols during storage may be one of the reasons for browning of RTE abalone. The results showed that the bloodletting treatment and adding antioxidants could delay the oxidation of phenols and thus reduce browning during processing and storage.

### 3.4. The Maillard Reaction in RTE Abalone Muscle during Storage

#### 3.4.1. Reducing Sugar Content

The changes in reducing sugar content in abalone muscle during storage are shown in Figure 2C. Before storage, the control group, bloodletting group and antioxidants group contained 7.81 ± 0.13, 7.89 ± 0.22 and 8.40 ± 0.35 mg/g of reducing sugar, respectively. After being stored for 40 days, the corresponding values dropped to 2.23 ± 0.24, 2.52 ± 0.13 and 3.87 ± 0.22 mg/g, respectively. Reducing sugar is an important substrate of the Maillard reaction, and its consumption during storage can reflect the severity of the Maillard reaction. The increase of pH value will increase the amount of open-chain reducing sugar, which is believed to promote the Maillard reaction [33]. Therefore, it was speculated that the addition of LA and CA inhibited the Maillard reaction and reduced reducing sugar consumption by decreasing pH value. In addition, the bloodletting treatment and adding antioxidants can delay the Maillard reaction by reducing metal ions and antioxidation. Chen et al. also found that the contents of polyphenols and reducing sugars decreased rapidly during storage, which led to the aggravation of non-enzymatic browning during storage [27].

#### 3.4.2. The Maillard Reaction Products

##### AGEs Fluorescence Intensity

**Figure 3:**
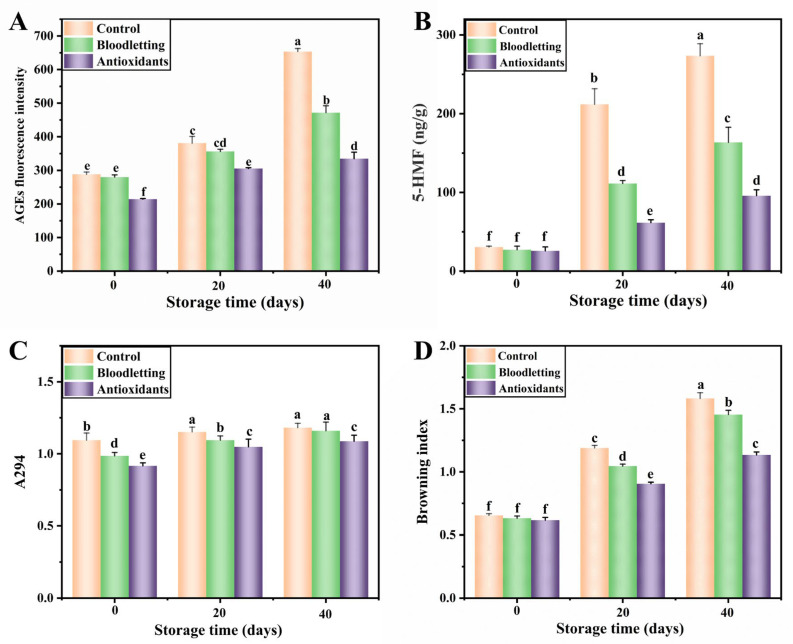
Changes in AGEs fluorescence intensity (**A**), 5-hydroxymethylfurfural content (**B**), Maillard intermediate product (A294) (**C**) and browning index (**D**) of abalone muscle during storage. Values of different groups with different lower-case letters are significantly different at *p* < 0.05.

The AGEs fluorescence products can effectively reflect the degree of the Maillard reaction during storage, which are mainly formed by the recombination of reducing compounds or amino compounds in the process of the Strecker degradation [34]. The changes in the AGEs fluorescence intensity of abalone muscle during storage are shown in Figure 3A. Before storage, the AGEs fluorescence intensity of RTE abalone in the antioxidants group was significantly lower than that of the others, which may be due to the fact that the Maillard reaction is controlled by antioxidants. The AGEs fluorescence intensity increased continuously during storage. After the period of storage, the order of fluorescence intensity of each group was as follows: antioxidants group, bloodletting group and control group. The fluorescence intensity of the control group was the highest probably because of the effect of Cu^2+^. Experiments of Hayase et al. [35] showed that Cu^2+^ can not only accelerate the formation of Amadori compounds but also affect their polymerization, leading to the formation of fluorescent compounds and products in the final stages of the Maillard reaction. At the same time, phenolic compounds can effectively reduce Maillard fluorescence, non-enzymatic browning, acrylamide and heterocyclic amines in different foods [34,36]. This confirms that the Maillard reaction in RTE abalone was effectively inhibited by bloodletting and adding antioxidants, with the antioxidants group having the best inhibitory effect.

##### 5-Hydroxymethylfurfural Content

Reducing sugars and amino acids could undergo the Maillard reaction to form a representative product, 5-hydroxymethyl-2-furfural (5-HMF), under acidic conditions [37]. Therefore, this six-carbon heterocyclic aldehyde is usually used as an indicator for the Maillard reaction [38]. The changes in the 5-HMF content in abalone muscle are shown in Figure 3B. Before storage, the 5-HMF contents in the control group, bloodletting group and antioxidants group were 30.62 ± 1.08, 27.13 ± 4.57 and 25.71 ± 5.02 ng/g, respectively. After 40 days of storage, the corresponding values rose to 273.45 ± 15.33, 163.45 ± 19.29 and 95.73 ± 7.61 ng/g, respectively. As mentioned above, the bloodletting treatment and addition of antioxidants can effectively inhibit the generation of 5-HMF by reducing the Cu^2+^ concentration, lowering pH and promoting antioxidant activity. Burdurlu and Karadeniz [39] showed that the browning index and lightness (L*) were related to 5-HMF concentration.

##### Maillard Intermediate Product (A294)

The absorbance at 294 nm reflected changes in the content of the Maillard intermediate compounds [40]. As shown in Figure 3C, after heat treatment, the content of A294 in the samples of each group was the highest in the control group and the lowest in the antioxidants group. In addition, the content of A294 in each group started rising from the beginning of storage, and in the antioxidants group, it was significantly lower than in the other groups. These results suggest that antioxidants can effectively reduce the promotion of Amadori product decomposition, and reduce the generation of colorless intermediates.

##### Browning Index (BI)

The brown nitrogenous polymers and copolymers, namely the BI, can be measured at a wavelength of 420 nm. As shown in Figure 3D, the BI of abalone muscle showed an increase over time during storage, which indicated the occurrence of the Maillard reaction. Obviously, after 40 days of storage, the order of the BI values of samples in each group was as follows: antioxidants group, bloodletting group and control group. Therefore, the BI in RTE abalone was effectively inhibited by bloodletting and adding antioxidants, with the antioxidants group having the best inhibitory effect. Our research team in the study of RTE shrimp also found that the BI value of shrimp increased during storage, and the addition of organic acids could effectively control the increase of BI [28].

### 3.5. Correlation Analysis of Various Factors

**Figure 4:**
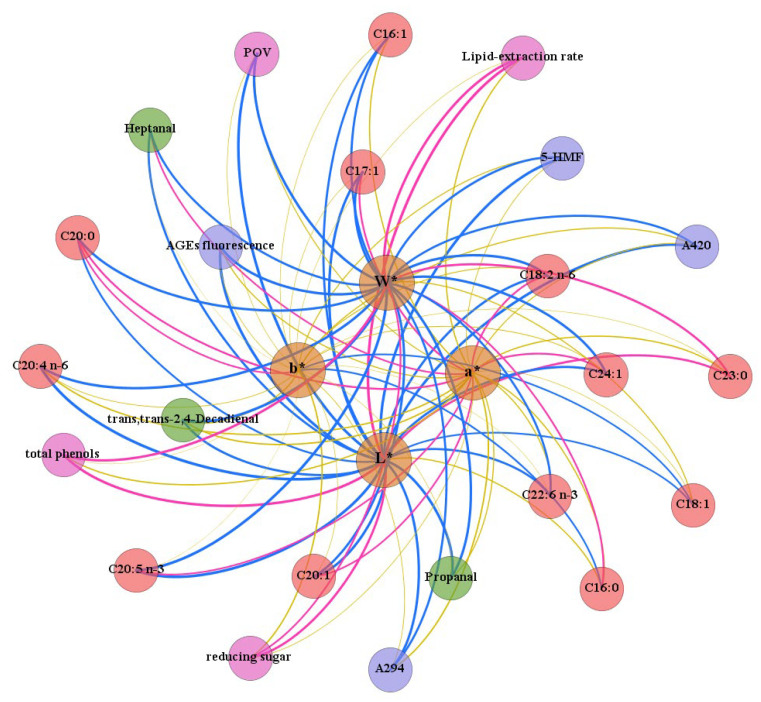
The correlation network between color parameters and related impact indicators of RTE abalone during storage. Brown nodes represent color parameters, while other nodes represent relevant impact indicators. The thickness of the line is proportional to the Pearson correlation value. The pink line and blue line indicate positive correlation and negative correlation, respectively, and the yellow line indicates weak correlation.

To further explore the correlation between color changes and various indicators, we conducted a network visualization analysis (Figure 4). A total of 27 nodes and 92 edges were observed in the network. The L* and W* values showed positive correlations with lipid-extraction rate, reducing sugar content and total phenols content, but negative correlations with the Maillard reaction products (AGEs fluorescence products, 5-HMF, Maillard intermediate product and BI), UFAs, POV and aldehydes production. Thus, the products of the Maillard reaction and oxidation of lipids and phenols were the reason for the reduction in the L* and W* values of RTE abalone. In other words, oxidation and the Maillard reaction lead to the browning of RTE abalone.

As can be seen in Table S1, the contents of UFAs, POV and aldehydes production are positively correlated with Maillard products, indicating that lipid oxidation and the Maillard reaction may promote each other. Based on the comparison of results reported in the literature and our findings, lipid oxidation products can modify the Maillard reaction by reacting with some intermediates or promoting the reaction to produce compounds different from those formed without lipids. The Maillard reaction products can not only promote but also reduce lipid oxidation. For instance, Amadori products have been shown to increase the oxidation of phospholipids [41]. At the same time, phenols and lipid oxidation also produce dark brown substances, affecting the color of RTE abalone.

## 4. Conclusions

The RTE abalone undergo browning during storage. Meanwhile, the content of reducing sugar, phenols and unsaturated fatty acids decreases, while the formation of lipid hydroperoxides and aldehydes increases during storage. Our research results indicated that the color change in RTE abalone during storage is mainly related to the Maillard reaction, while lipid oxidation may also take part in the Maillard reaction. Bloodletting can reduce Cu^2+^ in RTE abalone, and lactic acid, citric acid and 4-hexylresorcinol (LA, CA and 4-HR) can reduce the pH of RTE abalone and chelate metal ions, thereby inhibiting the occurrence of the Maillard reaction. Moreover, bloodletting and the addition of antioxidants can effectively slow down the oxidation and degradation of lipids in RTE abalone during storage, control the formation of aldehydes and thus inhibit the loss of nutrients in RTE abalone. Therefore, bloodletting and the addition of antioxidants can not only maintain the nutritional quality and color characteristics of abalone during processing, but also make the product more stable during storage. According to our research, bloodletting and the addition of antioxidants to RTE abalone have a good application prospect and popularizing value in the storage of RTE abalone and other RTE seafood. Even if this study explored the effects of non-enzymatic browning and lipid oxidation on the color of RTE abalone, what is the final color substance needs to be further discussed in future research.

## Figures and Tables

**Table 1:** Changes in the color of abalone muscle during storage.

Sample		L*	a*	b*	W*	ΔE
Control	0D	59.47 ± 1.96 ^ab^	6.91 ± 1.24 ^b^	37.80 ± 3.78 ^a^	44.15 ± 4.51 ^bc^	--
	20D	54.48 ± 5.32 ^c^	4.39 ± 1.51 ^d^	32.17 ± 1.84 ^a^	44.08 ± 2.98 ^bc^	62.95 ± 6.75 ^d^
	40D	44.80 ± 7.50 ^d^	4.88 ± 0.30 ^cd^	32.55 ± 4.95 ^a^	35.73 ± 3.02 ^d^	246.77 ± 14.18 ^a^
Bloodletting	0D	64.14 ± 3.79 ^a^	5.03 ± 0.88 ^c^	38.89 ± 4.39 ^a^	46.86 ± 3.88 ^b^	--
	20D	61.02 ± 3.21 ^ab^	2.70 ± 1.45 ^e^	34.46 ± 5.05 ^a^	47.90 ± 5.89 ^b^	34.78 ± 5.69 ^e^
	40D	52.58 ± 1.43 ^c^	8.17 ± 0.96 ^a^	36.30 ± 2.25 ^a^	39.72 ± 3.69 ^c^	150.21 ± 9.84 ^b^
Antioxidants	0D	65.16 ± 3.30 ^a^	2.83 ± 0.34 ^e^	33.14 ± 2.56 ^a^	51.84 ± 5.69 ^a^	--
	20D	63.25 ± 2.30 ^a^	3.33 ± 1.34 ^e^	29.87 ± 4.67 ^a^	52.52 ± 6.24 ^a^	14.62 ± 2.14 ^f^
	40D	56.95 ± 2.62 ^b^	4.35 ± 0.75 ^d^	29.40 ± 2.50 ^a^	47.69 ± 2.78 ^b^	83.86 ± 9.58 ^c^

Values in the same column with different lower-case letters (^a–f^) are significantly different at *p* < 0.05. L* (brightness), a* (+a, redness; −a, greenness), b * (+b, yellowness; −b, blueness), W* (whiteness) and ΔE (chromatic aberration).

**Table 2:** The fatty acid composition of RTE abalone muscle during storage (mg/g of dry basis).

Time (Days)		0			20			40	
Group	Control	Bloodletting	Antioxidants	Control	Bloodletting	Antioxidants	Control	Bloodletting	Antioxidants
C16:0	0.19 ± 0.02 ^a^	0.19 ± 0.02 ^a^	0.19 ± 0.02 ^a^	0.20 ± 0.02 ^a^	0.19 ± 0.02 ^a^	0.19 ± 0.01 ^a^	0.20 ± 0.02 ^a^	0.21 ± 0.01 ^a^	0.22 ± 0.02 ^a^
C16:1	2.60 ± 0.07 ^a^	2.63 ± 0.10 ^a^	2.70 ± 0.08 ^a^	2.27 ± 0.07 ^c^	2.49 ± 0.03 ^b^	2.45 ± 0.09 ^b^	2.11 ± 0.04 ^d^	2.32 ± 0.09 ^c^	2.31 ± 0.06 ^c^
C17:1	0.75 ± 0.01 ^ab^	0.78 ± 0.01 ^a^	0.81 ± 0.01 ^a^	0.65 ± 0.03 ^b^	0.73 ± 0.01 ^ab^	0.81 ± 0.01 ^a^	0.58 ± 0.02 ^c^	0.63 ± 0.02 ^b^	0.73 ± 0.02 ^ab^
C18:1	0.48 ± 0.01 ^b^	0.53 ± 0.02 ^ab^	0.58 ± 0.01 ^a^	0.48 ± 0.03 ^b^	0.50 ± 0.02 ^ab^	0.52 ± 0.04 ^ab^	0.41 ± 0.02 ^c^	0.52 ± 0.03 ^ab^	0.68 ± 0.01 ^ab^
C18:2 n-6	3.94 ± 0.13 ^ab^	3.98 ± 0.09 ^ab^	4.10 ± 0.09 ^a^	3.36 ± 0.06 ^c^	3.59 ± 0.15 ^b^	3.89 ± 0.08 ^ab^	2.71 ± 0.09 ^d^	2.81 ± 0.11 ^d^	3.59 ± 0.11 ^b^
C20:0	1.13 ± 0.04 ^a^	1.11 ± 0.08 ^a^	1.13 ± 0.06 ^a^	1.09 ± 0.03 ^a^	1.11 ± 0.04 ^a^	1.13 ± 0.01 ^a^	1.09 ± 0.04 ^a^	1.06 ± 0.09 ^a^	1.08 ± 0.03 ^a^
C20:1	0.55 ± 0.01 ^a^	0.56 ± 0.01 ^a^	0.59 ± 0.02 ^a^	0.48 ± 0.01 ^b^	0.54 ± 0.01 ^a^	0.59 ± 0.03 ^a^	0.46 ± 0.02 ^b^	0.52 ± 0.02 ^a^	0.54 ± 0.02 ^a^
C20:4 n-6	1.06 ± 0.03 ^b^	1.11 ± 0.03 ^ab^	1.16 ± 0.02 ^a^	1.01 ± 0.02 ^bc^	1.05 ± 0.02 ^b^	1.11 ± 0.04 ^ab^	0.99 ± 0.03 ^bc^	1.04 ± 0.04 ^b^	1.06 ± 0.01 ^b^
C20:5 n-3	0.88 ± 0.03 ^b^	0.91 ± 0.01 ^b^	1.00 ± 0.02 ^a^	0.83 ± 0.02 ^bc^	0.86 ± 0.02 ^b^	0.94 ± 0.03 ^ab^	0.77 ± 0.02 ^c^	0.79 ± 0.04 ^c^	0.90 ± 0.03 ^b^
C22:6 n-3	1.05 ± 0.03 ^b^	1.14 ± 0.02 ^a^	1.11 ± 0.03 ^a^	1.01 ± 0.03 ^b^	1.03 ± 0.04 ^b^	1.03 ± 0.03 ^b^	0.91 ± 0.02 ^c^	0.98 ± 0.02 ^bc^	1.01 ± 0.02 ^b^
C23:0	1.52 ± 0.01 ^a^	1.53 ± 0.02 ^a^	1.49 ± 0.02 ^a^	1.54 ± 0.02 ^a^	1.51 ± 0.04 ^a^	1.52 ± 0.06 ^a^	1.60 ± 0.02 ^a^	1.55 ± 0.04 ^a^	1.52 ± 0.06 ^a^
C24:1	0.37 ± 0.01 ^a^	0.38 ± 0.01 ^a^	0.38 ± 0.02 ^a^	0.34 ± 0.01 ^ab^	0.36 ± 0.01 ^a^	0.38 ± 0.02 ^a^	0.31 ± 0.04 ^b^	0.33 ± 0.02 ^ab^	0.35 ± 0.02 ^a^
SFAs	2.85 ± 0.15 ^a^	2.83 ± 0.13 ^a^	2.81 ± 0.16 ^a^	2.82 ± 0.06 ^a^	2.81 ± 0.21 ^a^	2.84 ± 0.15 ^a^	2.89 ± 0.14 ^a^	2.81 ± 0.19 ^a^	2.82 ± 0.15 ^a^
MUFAs	4.74 ± 0.21 ^b^	4.88 ± 0.19 ^ab^	5.05 ± 0.23 ^a^	4.21 ± 0.25 ^c^	4.62 ± 0.23 ^bc^	4.74 ± 0.14 ^b^	3.88 ± 0.26 ^d^	4.32 ± 0.21 ^c^	4.60 ± 0.31 ^bc^
PUFAs	6.92 ± 0.19 ^b^	7.14 ± 0.23 ^ab^	7.37 ± 0.21 ^a^	6.21 ± 0.18 ^d^	6.53 ± 0.16 ^c^	6.97 ± 0.19 ^b^	5.38 ± 0.16 ^f^	5.62 ± 0.23 ^e^	6.55 ± 0.21 ^c^

Values in the same line with different lower-case letters (^a–e^) are significantly different at *p* < 0.05. Abbreviations are as follows: SFAs, saturated fatty acids; MUFAs, monounsaturated fatty acids; PUFAs, polyunsaturated fatty acids.

**Table 3:** The contents of aldehydes in RTE abalone muscle during storage (µg/g of lipid).

Time (Days)		0			20			40	
Group	Control	Bloodletting	Antioxidants	Control	Bloodletting	Antioxidants	Control	Bloodletting	Antioxidants
Propanal	32.50 ± 1.52 ^c^	13.31 ± 1.90 ^f^	11.77 ± 2.56 ^f^	37.67 ± 0.07 ^b^	23.19 ± 1.99 ^d^	15.42 ± 1.49 ^f^	55.47 ± 2.34 ^a^	26.15 ± 0.79 ^d^	21.40 ± 2.06 ^e^
Heptanal	16.12 ± 0.01 ^a^	6.70 ± 0.36 ^c^	6.02 ± 0.03 ^d^	11.96 ± 0.63 ^b^	8.90 ± 0.71 ^c^	9.52 ± 0.24 ^c^	16.64 ± 2.15 ^a^	11.61 ± 4.23 ^b^	10.61 ± 1.54 ^b^
trans,trans-2,4-Decadienal	200.21 ± 2.09 ^e^	232.58 ± 5.62 ^d^	125.02 ± 10.94 ^g^	261.96 ± 4.35 ^c^	260.23 ± 1.00 ^c^	124.36 ± 5.62 ^g^	337.08 ± 8.65 ^a^	296.5 ± 19.13 ^b^	161.67 ± 31.11 ^f^

Values in the same line with different lower-case letters (^a–g^) are significantly different at *p* < 0.05.

## Data Availability

Data are contained within the article.

## Supplementary Materials

The following supporting information can be downloaded at: https://www.mdpi.com/article/10.3390/foods12071514/s1, Table S1: The correlation coefficients (*r*) and probabilities (*P*) of significance of RTE abalone during storage.

## References

[1] Food and Agriculture Organization of the United Nations FIGIS List of Species for Fishery Global Production Statistics. http://www.fao.org/fishery/statistics/en.

[2] Chiou T., Tsai C., Lan H. (2004). Chemical, physical and sensory changes of small abalone meat during cooking. Fish. Sci..

[3] Singh A., Benjakul S. (2017). Effect of serine protease inhibitor from squid ovary on gel properties of surimi from Indian mackerel. Texture Stud..

[4] Jiao S., Zhu D., Deng Y., Zhao Y.Y. (2016). Effects of hot air-assisted radio frequency heating on quality and shelf-life of roasted peanuts. Food Bioproc. Technol..

[5] Wang X.F., Cong H., Wang H.B. (2011). Research review of snack and cooked fish food. Wuhan Polytech. Univ..

[6] Nunak N., Schleining G. (2011). Instrumental textural changes in raw white shrimp during iced storage. Aquat. Food Prod. Technol..

[7] Dong X.P., Hou Y.W., Wang Y., Xu X.B., Wang K.X., Zhao M.Y., Prakash S., Yu C.X. (2018). Effect of temperature-time pretreatments on the texture and microstructure of abalone (*Haliotis discus hanai*). Texture Stud..

[8] Friedman M. (1996). Food browning and its prevention: An overview. J. Agric. Food Chem..

[9] Li D.Y., Liu Z.Q., Liu B., Qi Y., Liu Y.X., Liu X.Y., Qin L., Zhou D.Y., Shahidi F. (2020). Effect of protein oxidation and degradation on texture deterioration of ready-to-eat shrimps during storage. J. Food Sci..

[10] Mozuraityte R., Rustad T., Storrø I. (2010). Pro-oxidant activity of Fe^2+^ in oxidation of cod phospholipids in liposomes. Eur. J. Lipid Sci. Technol..

[11] Wang Y., Hui T., Zhang Y.W., Liu B., Wang F.L., Li J.K., Cui B.W., Guo X.Y., Peng Z.Q. (2015). Effects of frying conditions on the formation of heterocyclic amines and trans fatty acids in grass carp (*Ctenopharyngodon idellus*). Food Chem..

[12] Burmester T. (2002). Origin and evolutiong of arthropod hemocyanin and related proteins. Comp Physiol..

[13] Jaenicke E., Decker H. (2004). Conversion of crustacean hemocyanin to catecholoxidase. Micron.

[14] Dong X., Zhang Y.L., Wang F., Pang M.X., Qi J.H. (2016). Relationship in between Chemical Oxidation and Browning of Flavanols. IOP Conf. Ser. Earth Environ. Sci..

[15] Lian F., Måge I., Lorentzen G., Siikavuopio S.I., Øverbø K., Vang B., Lindberg D. (2018). Exploring the effect of inhibitors, cooking and freezing on melanosis in snow crab (*Chionoecetes opilio*) clusters. Food Control.

[16] Martínez-Alvarez O., López-Caballero M.E., Montero P., Gómez-Guillén M.D.C. (2020). The effect of different melanosis-inhibiting blends on the quality of frozen deep-water rose shrimp (*Parapenaeus longirostris*). Food Control.

[17] Skipnes D., Johnsen S.O., Skåra T., Sivertsvik M., Lekang O. (2011). Optimization of heat processing of farmed Atlantic cod (*Gadus morhua*) muscle with respect to cook loss, water holding capacity, colour, and texture. J. Aquat. Food Prod. Technol..

[18] Folch J., Lees M., Stanley G.H.S. (1957). A simple method for the isolation and purification of total lipides from animal tissues. J. Biol. Chem..

[19] Xie H.K., Zhou D.Y., Hu X.P., Liu Z.Y., Song L., Zhu B.W. (2018). Changes in lipid profiles of dried clams (*Mactra chinensis* Philippi and *Ruditapes philippinarum*) during accelerated storage and prediction of shelf life. J. Agric. Food Chem..

[20] Xie H.K., Zhou D.Y., Liu Z.Y. (2019). Effects of natural phenolics on shelf life and lipid stability of freeze-dried scallop adductor muscle. Food Chem..

[21] Zhou F.Z., Zeng T., Yin S.W., Tang C.H., Yuan D.B., Yang X.Q. (2018). Development of antioxidant gliadin particle stabilized Pickering high internal phase emulsions (HIPEs) as oral delivery systems and the in vitro digestion fate. Food Funct..

[22] Zhao G.H., Hu Y.Y., Liu Z.Y. (2021). Simultaneous quantification of 24 aldehydes and ketones in oysters (*Crassostrea gigas*) with different thermal processing procedures by HPLC-electrospray tandem mass spectrometry. Food Res. Int..

[23] Zhao M.T., Liu Z.Y., Li A., Zhao G.H., Xie H.K., Zhou D.Y., Wang T. (2021). Gallic acid and its alkyl esters emerge as effective antioxidants against lipid oxidation during hot air drying process of *Ostrea talienwhanensis*. LWT Food Sci. Technol..

[24] Matiacevich S.B., Pilar Buera M. (2006). A critical evaluation of fluorescence as a potential marker for the Maillard reaction. Food Chem..

[25] Serra-Cayuela A., Jourdes M., Riu-Aumatell M., Buxaderas S., Teissedre P.L., López-Tamames E. (2014). Kinetics of browning, phenolics, and 5-hydroxymethylfurfural in commercial sparkling wines. J. Agric. Food Chem..

[26] Ajandouz E.H., Tchiakpe L.S., Ore F.D., Benajiba A., Puigserver A. (2001). Effects of pH on caramelization and Maillard reaction kinetics in fructose-lysine model systems. J. Food Sci..

[27] Wang C., Zhang X., Gao Y. (2020). Path analysis of non-enzymatic browning in Dongbei Suancai during storage caused by different fermentation conditions. Food Chem..

[28] Li D.Y., Li N., Dong X.H. (2022). Effect of phytic acid combined with lactic acid on color and texture deterioration of ready-to-eat shrimps during storage. Food Chem..

[29] Uematsu T., Parkányiová L., Endo T., Matsuyama C., Pokorný J. (2002). Effect of the unsaturation degree on browning reactions of peanut oil and other edible oils with proteins under storage and frying conditions. Int. Congr..

[30] Wang Z.M., He Z.F., Gan X. (2018). Interrelationship among ferrous myoglobin, lipid and protein oxidations in rabbit meat during refrigerated and superchilled storage. Meat Sci..

[31] Cyprian O.O., Nguyen M.V., Sveinsdottir K. (2017). Influence of blanching treatment and drying methods on the drying characteristics and quality changes of dried sardine (*Sardinella gibbosa*) during storage. Dry. Technol..

[32] Li D.Y., Xie H.K., Liu Z., Li A., Li J., Liu B. (2019). Shelf life prediction and changes in lipid profiles of dried shrimp (Penaeus vannamei) during accelerated storage. Food Chem..

[33] Lertittikul W., Benjakul S., Tanaka M. (2007). Characteristics and antioxidative activity of Maillard reaction products from a porcine plasma protein-glucose model system as influenced by pH. Food Chem..

[34] Rannou C., Laroque D., Renault E. (2016). Mitigation strategies of acrylamide, furans, heterocyclic amines and browning during the Maillard reaction in foods. Food Res. Int..

[35] Hayase F., Shibuya T., Sato J., Yamamoto M. (1996). Effect of oxygen and transition metals on the advanced Maillard reaction of proteins with glucose. Biosci. Biotechnol. Biochem..

[36] Demirok E., Kolsarici N. (2014). Effect of green tea extract and microwave pre-cooking on the formation of acrylamide in fried chicken drumsticks and chicken wings. Food Res. Int..

[37] Liu S.C., Huang M.C., James S.W. (2003). A study on the mechanism of browning in mei liqueur using model solutions. Food Res. Int..

[38] Kowalski S., Lukasiewicz M., Duda-Chodak A., Zięć G. (2013). 5-Hydroxymethyl-2-furfural (HMF)–heat-induced formation, occurrence in food and biotransformation—A review. Pol. J. Food Nutr. Sci..

[39] Burdurlu H.S., Karadeniz F. (2003). Effect of storage on nonenzymatic browning of apple juice concentrates. Food Chem..

[40] Yu P., Xu X.B., Yu S.J. (2017). The effect of pH and amino acids on the formation of methylglyoxal in a glucose-amino acid model system. J. Sci. Food Agric..

[41] Zamora R., Hidalgo F.J. (2011). The Maillard reaction and lipid oxidation. Lipid Technol..

