# Supplementary material for: Effects of Non-Enzymatic Browning and Lipid Oxidation on Color of Ready-to-Eat Abalone during Accelerated Storage and Its Control
Source: Foods. 2023 Apr 3;12(7):1514. doi: 10.3390/foods12071514 (PMC10094519; doi:10.3390/foods12071514)
Supplement: Supplementary file 1 [file foods-12-01514-s001.zip › foods-2277271-supplementary.pdf]

Supplementary Materials

Table S1. The correlation coefficients (*r*) and probabilities (*P*) of significance of RTE abalone during storage.

	L*	a*	b*	W*	C16:0	C16:1	C17:1	C18:1	C18:2 n-6	C20:0	C20:1	C23:0	C20:4 n-6	C20:5 n-3	C24:1	C22:6 n-3	Propanal	Heptanal	trans,trans-2,4-Decadienal	A294	A420	AGEs	5-HMF	Oil-extraction rate	POV	total phenols content	reducing sugar content
L*	1.00	-0.43	0.16	0.91**	0.36	-0.75*	-0.86**	-0.55	-0.79*	-0.64	-0.87**	0.73*	-0.97**	-0.96**	-0.80**	-0.74*	-0.90**	-0.77*	-0.78*	-0.84**	-0.90**	-0.95**	-0.92**	0.92**	-0.93**	0.87**	0.79*
a*		1.00	0.54	-0.64	-0.35	0.37	0.60	0.09	0.60	0.63	0.55	-0.39	0.43	0.53	0.61	0.32	0.30	0.53	0.44	0.45	0.27	0.32	0.16	-0.37	0.26	-0.40	-0.14
b*			1.00	-0.27	-0.58	0.09	0.09	-0.55	0.25	0.52	-0.09	-0.07	-0.16	0.01	0.19	0.03	-0.07	0.01	0.34	-0.15	-0.33	-0.12	-0.28	0.07	-0.10	0.01	0.43
W*				1.00	0.58	-0.76*	-0.87**	-0.31	-0.87**	-0.83**	-.080**	0.74*	-0.88**	-0.94**	-0.85**	-0.73*	-0.85**	-0.75*	-0.90**	-0.74*	-0.74*	-0.87**	-0.77*	0.86**	-0.87**	0.83**	0.58
C16:0					1.00	-0.63	-0.49	0.48	-0.54	-0.84**	-0.40	0.30	-0.43	-0.39	-0.59	-0.51	-0.53	-0.39	-0.71*	-0.36	-0.23	-0.39	-0.41	0.50	-0.44	0.61	0.18
C16:1						1.00	0.85**	0.28	0.87**	0.72*	0.84**	-0.81**	0.85**	0.79*	0.91**	0.92**	0.90**	0.87**	0.74*	0.87**	0.58	0.70*	0.72*	-0.90**	0.72*	-0.82**	-0.60
C17:1							1.00	0.50	0.95**	0.76*	0.95**	-0.86**	0.91**	0.95**	0.98**	0.83**	0.80**	0.78*	0.82**	0.92**	0.79*	0.78*	0.78*	-0.88**	0.72*	-0.89**	-0.77*
C18:1								1.00	0.42	-0.08	0.53	-0.61	0.51	0.58	0.39	0.39	0.36	0.38	0.18	0.56	0.59	0.46	0.46	-0.44	0.37	-0.32	-0.63
C18:2 n-6									1.00	0.81**	0.84**	-0.84**	0.82**	0.91**	0.97**	0.88**	0.81**	0.78*	0.82**	0.82**	0.61	0.68*	0.68*	-0.88**	0.66	-0.78*	-0.59
C20:0										1.00	0.65	-0.55	0.63	0.72*	0.79*	0.58	0.70*	0.67	0.79*	0.55	0.44	0.58	0.51	-0.67*	0.63	-0.697*	-0.31
C20:1											1.00	-0.83**	0.93**	0.89**	0.92**	0.76*	0.79*	0.81**	0.72*	0.98**	0.85**	0.82**	0.79*	-0.83**	0.74*	-0.90**	-0.82**
C23:0												1.00	-0.77*	-0.84**	-0.85**	-0.76*	-0.72*	-0.72*	-0.75*	-0.85**	-0.66	-0.74*	-0.63	0.73*	-0.67*	0.74*	0.66
C20:4 n-6													1.00	0.94**	0.88**	0.83**	0.91**	0.80**	0.79*	0.93**	0.90**	0.92**	0.93**	-0.95**	0.89**	-0.93**	-0.84**
C20:5 n-3														1.00	0.89**	0.79*	0.87**	0.78*	0.84**	0.86**	0.84**	0.89**	0.84**	-0.91**	0.86**	-0.86**	-0.75*
C24:1															1.00	0.89**	0.80**	0.78*	0.84**	0.91**	0.70*	0.72*	0.74*	-0.89**	0.66	-0.88**	-0.71*
C22:6 n-3																1.00	0.84**	0.71*	0.76*	0.83**	0.60	0.66	0.78*	-0.94**	0.64	-0.79*	-0.66
Propanal																	1.00	0.87**	0.74*	0.79*	0.69*	0.84**	0.84**	-0.95**	0.91**	-0.80**	-0.61
Heptanal																		1.00	0.52	0.78*	0.52	0.66	0.57	-0.79*	0.73*	-0.63	-0.43

trans,trans-2,4-Decadienal	1.00	0.70*	0.71*	0.83**	0.78*	-0.79*	0.77*	-0.88**	-0.64
A294		1.00	0.83**	0.79*	0.81**	-0.85**	0.70*	-0.90**	-0.86**
A420			1.00	0.91**	0.92**	-0.76*	0.81**	-0.90**	-0.94**
AGEs				1.00	0.91**	-0.82**	0.96**	-0.88**	-0.78*
5-HMF					1.00	-0.90**	0.87**	-0.91**	-0.89**
Oil-extraction rate						1.00	-0.83**	0.87**	0.74*
POV							1.00	-0.80**	-0.65
total phenols content								1.00	0.86**
reducing sugar content									1.00

* $P < 0.05$. ** $P < 0.01$.
